# Protective Effects of Dexrazoxane against Doxorubicin-Induced Cardiotoxicity: A Metabolomic Study

**DOI:** 10.1371/journal.pone.0169567

**Published:** 2017-01-10

**Authors:** Yang QuanJun, Yang GenJin, Wan LiLi, Han YongLong, Huo Yan, Li Jie, Huang JinLu, Lu Jin, Gan Run, Guo Cheng

**Affiliations:** 1 Department of Pharmacy, Shanghai Jiao Tong University Affiliated Sixth People’s Hospital, Shanghai, China; 2 School of Pharmacy, Second Military Medical University, Shanghai, China; Virginia Commonwealth University, UNITED STATES

## Abstract

Cardioprotection of dexrazoxane (DZR) against doxorubicin (DOX)-induced cardiotoxicity is contentious and the indicator is controversial. A pairwise comparative metabolomics approach was used to delineate the potential metabolic processes in the present study. Ninety-six BALB/c mice were randomly divided into two supergroups: tumor and control groups. Each supergroup was divided into control, DOX, DZR, and DOX plus DZR treatment groups. DOX treatment resulted in a steady increase in 5-hydroxylysine, 2-hydroxybutyrate, 2-oxoglutarate, 3-hydroxybutyrate, and decrease in glucose, glutamate, cysteine, acetone, methionine, asparate, isoleucine, and glycylproline.DZR treatment led to increase in lactate, 3-hydroxybutyrate, glutamate, alanine, and decrease in glucose, trimethylamine N-oxide and carnosine levels. These metabolites represent potential biomarkers for early prediction of cardiotoxicity of DOX and the cardioprotective evaluation of DZR.

## Introduction

Doxorubicin (DOX) is one of the most effective and widely used anticancer drugs. Its dose-dependent anticancer activity was discovered over forty years ago[1]. Decreased dosage suggests reduced anticancer efficacy and poor survival. However, curative doses often relate to severe cardiotoxicity, including life-threatening cardiomyopathy and congestive heart failure[2, 3]. The prevalence of DOX-induced heart failure was estimated at 5%, 26%, and 48% in patients at cumulative doses up to 400, 550, and 700 mg/m^2^, respectively[4]. Several potential mechanisms of DOX-induced cardiotoxicity were suggested, and after comprehensive basic and clinical investigation, the free radical hypothesis was acknowledged[5–7]. Dexrazoxane(DZR) mediates EDTA-like hydrolysis, resulting in chelating iron and decreased level of hydroxyl free radicals, and therefore, was clinically approved for protection against cardiotoxicity at cumulative doses of DOX up to 300 mg/m^2^.

However, DOX-induced cardiotoxicity occurs even at doses less than 240 mg/m^2^[8] at any point during and subsequent to treatment, and even progress to late-onset cardiotoxicity. Further, DOX-induced delayed cardiotoxicity occurs without an acute or early-onset phase [9]. Measures to predict DOX-induced cardiotoxicity and signs of DZR treatment in patients with DOX-based chemotherapy regimens need to be developed. Traditionally, monitoring of cardiac function during DOX therapy was based on left ventricular ejection fraction (LVEF), which failed to detect changes in DOX-induced subclinical cardiotoxicity[10, 11]. Serum biomarkers including cardiac troponin T (cTnT) also do not specifically reflect cardiac damage[12–14]. New biomarkers are, therefore, needed for the evaluation of cardiac damage and indications for DZR treatment.

DOX-induced myocardial free radical oxidative stress and DZR-related hydrolysis alter a range of biochemical parameters, which affect downstream metabolic processes[15–18]. Detection of altered metabolites earlier than the myocardial injury represents a potential biomarker for early diagnosis of DOX-induced cardiotoxicity and prediction of DZR treatment outcomes. Metabolomics is an unbiased global approach that reveals all the disease-related biological changes and drug-induced aberrations. It is widely used for the identification of biomarkers for pathological diagnosis and toxicity prediction[19, 20].

In the present study, pairwise comparative metabolomics was employed to reveal the potential metabolic processes following DOX and DZR treatment. Due to the metabolite diversity, the ^1^H-based high resolution NMR-based analysis was used to detect altered serum metabolites. To reduce variation between subjects and optimize data recovery, the experimental mice were divided into tumor-bearing and control supergroups, and each supergroup was divided into control, DOX, and DZR treatment groups, and DOX+DZR co-treatment subgroups. The results indicate potential biomarkers for early evaluation of DOX-induced cardiotoxicity and shed light on the metabolic mechanism underlying DZR-related cardioprotective effects.

## Materials and Methods

### Materials and reagents

DZR hydrochloride was purchased from Jiangsu Aosaikang Pharmaceutical Co, Ltd, (Jiangsu, China). DOX hydrochloride (adriamycin) was purchased from Shenzhen Arcandor's Pharmaceutcal Co., LTD (Shenzhen, China). CT26 colorectal carcinoma cells were obtained from the Typical Culture Preservation Commission Cell Bank, Chinese Academy of Sciences (Shanghai, China).Creatine kinase (CK) and creatine kinase myocardial bound (CK-MB), lactate dehydrogenase (LDH) and toal glutathione / oxidized glutathione assay kits were purchased from Nanjing Jiancheng Bioengineering Institute (Jiangsu, China). The mouse cardiac troponin T (cTnT) enzyme linked immunosorbent assay (ELISA) kits were from Sincere Biotech Co., Ltd (Beijing, China).

### Animals

Male BALB/c mice were purchased from Shanghai SLAC Laboratory Animal Co. Ltd. (Shanghai, China). All procedures involving animals and their care were approved by the animal care committee of Shanghai Jiao Tong University Affiliated Sixth People’s Hospital in accordance with the Chinese government guidelines for animal experiments. The facility is under the supervision of the local representative of the animal welfare agency. The mice were maintained under specific pathogen-free conditions. All mice were exposed to a 12-hour light/dark cycle, and fed a commercial standard diet, with water *adlibitum*. All efforts were made to ameliorate animal suffering and their health were monitored every day during the experimental procedure. According to our laboratory protocol, the clinical signs used to determine when to euthanize the animals is weight loss > 20%.

### Animal model and experimental design

**Fig 1:**
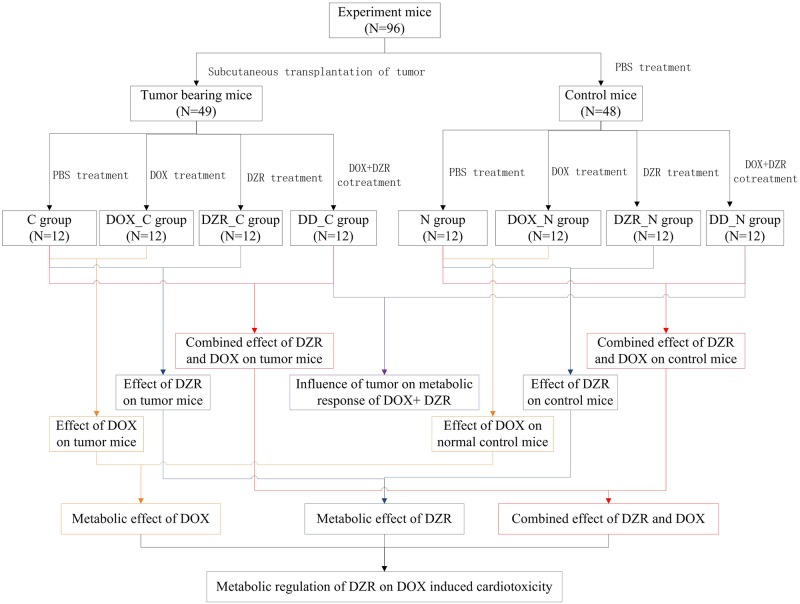
Flowchart outlining the study design and experimental process. Pairwise analysis was showed as the same color and broken line indicated the corresponding groups were not included in the pairwise analysis.

A CT26 colorectal carcinoma subcutaneous transplantation model was established as previously described[21]. Briefly, 96 BALB/c mice were randomly divided into two supergroups. Forty-eight mice were subcutaneously injected with 10^6^ CT26 cells(suspension) in their left flank, which represented the tumor-bearing supergroup. The other forty-eight mice treated with an equivalent volume of phosphate buffer solution (PBS) served as the control. The day of transplantation of cancer cells day 0. The tumor was first palpable on day 7 and then the greatest longitudinal diameter (length in cm) and the greatest transverse diameter (width in cm) of tumor were measured using a digital caliper. In vivo tumor volume (in cm^3^) were calculated by the modified ellipsoidal formula as tumor volume = 1/2(length × width ^2^). The mice in each supergroup were randomly divided into four subgroups (12 mice each): control, DOX, DZR, and DOX+DZR treatment (Fig 1). DOX mice were intraperitoneally injected with 0.1 ml DOX solution (12mg/kg/day, dissolved in double-distilled water) on days 8, 11 and 14. DZR mice were intraperitoneally injected with 0.1 ml DZR solution (200 mg/kg/day, dissolved in 0.167mol/L sodium lactate solution). DOX+DZR mice were consecutively injected with DZR and DOX solution at intervals of 3 h. The control mice were intraperitoneally injected with 0.1 ml sodium lactate solution, correspondingly. On day 15, all the mice were euthanized by carbon dioxide inhalation. Blood was collected into non-anticoagulant tubes. The heart was quickly washed with pre-cooled PBS, weighed, and transected into two sections parallel to the atrioventricular sulcus. The left ventricular wall was fixed in 10% neutral formalin solution and the other fragment was immediately preserved in liquid nitrogen. The lung, liver, spleen and kidney were dissected, weighed and frozen in liquid nitrogen. The tumors were removed and the carcass weight was measured.

### Detection of myocardial pathology and cardiac injury markers

The fixed left ventricular walls were embedded in paraffin according to standard procedures. The paraffin blocks were serially sectioned, dewaxed, and stained with haematoxylin-eosin as conventional protocols. The specimens were dehydrated in alcohol gradient, cleared, and mounted. Every heart tissue slide was analyzed by two pathologists. Results were recorded as the median of two independent scores for each animal. Blood samples were intravenously collected and serum was separated by centrifugation for determination of CK and CK-MB level, while heart tissue was homogenized and myocardial LDH, cTNT, total glutathione and oxidized glutathione were measured according to standard methods using diagnostic kits (Nanjing Jiancheng Biological Product, China). Briefly, 50 mg myocardial tissue was homogenized and diluted to 0.25% with ice-cold 40 mM Tris–HCl buffer (pH 7.4). The samples were then centrifuged at 12000 rpm for 20 min at 4°C and the supernatant was collected. The supernatant was incubated with corresponding reagent and the fluorescence intensity was measured by BioTek Epoch Microplate Spectrophotometer (Biotek, Winooski, VT, USA).

### Serum^1^H-NMR spectroscopy

All the spectra were recorded using a Bruker AMX-600 NMR spectrometer operated at a 600.13 MHz 1H resonance frequency. The serum samples were prepared for NMR analysis by mixing 200 μL of serum with 400 μL of PBS (containing 10% v/v D_2_O). A solvent pre-saturation was also used to suppress the water peaks. To attenuate the broad NMR signals from slowly tumbling molecules, a standard Carr-Purcell-Meiboom-Gill (CPMG) pulse sequence was used to record the 1D spin-echo spectra. Briefly, the sequence was—RD—90° - (*t—*180° - *t*)_*n*_*—*ACQ, where RD represents the relaxation delay of 2 s, 90° and 180° represent the RF pulses that trip the magnetization vector, *t* is the spin-echo delay of 400 μs, *n* is the loop number of 80, and ACQ is the data acquisition period of 1.36 s[22]. The data points were acquired using 128 transients in our experiment, and the number of time domain points was 32k. The quality control tests for 1H-NMR spectroscopy were performed at the beginning of every measurement day. A representative sample was used for NMR probe tuning and matching, determination of the transmitter offset value for water pulse presaturation and 90 pulse adjustments.

### Reduction of NMR data

The corrected NMR spectra corresponding to the chemical shift range of δ 0.2–10.0 were imported into AMIX 3.9.5 (Bruker Biospin, Rheinstetten, Germany). All the spectra were reduced into integral regions of equal length of 0.01 ppm. Regions of δ 4.7–5.1 that contained the resonance from residual water were set to zero. The data were normalized to the total spectral area (100%) to reduce the concentration differences between the samples.

### Metabolomics analysis

The dataset was analyzed using pattern recognition methods in the software Simca-P 11.5 (UmetricsAB, Umeå, Sweden). The dataset was arranged with the samples as observations and the peak areas of the chemical shifts represented the response variables. Before multivariate analysis, the response variables were centered and scaled to Pareto variance, the base weight was computed as 1/sqrt, and data without normal distribution were log transformed.

To explain the maximum variation between the samples, a principal component analysis (PCA) bilinear decomposition method was used to view the clusters within the multivariate data. A Partial Least Squares-Discriminate Analysis (PLS-DA) was employed to explain the maximum separation between the defined class samples in the dataset. Three parameters, R^2^X, R^2^Y and Q^2^Y, were used for the evaluation of the models. R^2^X explains the cumulative variation in the response variables. R^2^Y represents the latent variable of the sum of squares of all of the Xs and Ys. Q^2^ reflects the cumulative cross-validated percentage of the total variation that can be predicted by the current latent variables. High coefficient values of R^2^Y and Q^2^Y represent good discrimination and predictive ability[19, 23]. The specific metabolites between classes were interpreted using variable importance in the projection (VIP) and correlation coefficients. Variables with a high VIP were considered statistically significant.

### Metabolite identification

Based on the statistical results of the metabolomics analysis, the discriminating peaks were prioritized for identification. The NMR signals were compared with reference spectra from the HMDB database and the Chenomx NMR Suite metabolomics software (Chenomx, Inc., Alberta, Canada) based on the coupling constant and the spitting model. The signal assignments were carried out using the 2D J-Resolved (jresgpprqf), COSY (cosygpprqf), and TOCSY (mlevphpr) spectra.

### Statistical methods

Data were expressed as the mean ± SD. The calculated means were statistically analyzed using GraphPad Prism, version 5.0 (GraphPad Software Inc., San Diego, CA). Differences involving more than two groups were compared using one-way ANOVA, followed by Tukey's post-hoc test. The differences between the two groups were analyzed using a two-sided Student’s t-test. The level of significance was set at *p*< 0.05.

## Results

### DZR partially prevented DOX-induced LBW, tumor and organ weight loss

The mean baseline body weight of all 96 mice used was 20.31 ± 0.94 g. No differences among eight groups were observed in the present study. During the post-treatment period, two mice died in the DOX-treated cancer group and one died in the DOX-treated control group without intervention. These might due to the toxicity of the DOX. No deaths occurred in the other groups. The dynamic tumor volume over the course of your experiment was showed in S1 Fig. The LBW, tumor and organ weights at the time of sacrifice are listed in Table 1. DOX treatment resulted in significant reduction of LBW when compared with the matched control groups. DZR partially prevented DOX-induced LBW loss when administered as DOX+DZR combination therapy, and mice showed an intermediate lean body weight. DOX treatment also caused a significant reduction in heart weight in both the cancer and normal control groups compared with matched control groups. DOX+DZR treated animals showed increased body weight when compared with the corresponding DOX alone treated animals. Further, the ratios of heart weight to lean body weight were significantly decreased in the DOX-treated mice when compared with matched control groups, whereas in the other groups the reduction in heart mass correlated with the decrease in lean body weight.

**Table 1:** Effect of DOX and DZR treatment on body and organ weights.

	Tumor-Bearing Mice				Control Mice			
	C (n = 12)	DOX_C (n = 10)	DZR_C (n = 12)	DD_C (n = 12)	N (n = 12)	DOX_N (n = 11)	DZR_N (n = 12)	DD_N (n = 12)
Tumor(g)	1.42±0.26	0.95±0.31	1.46±0.61	0.97±0.58d				
LBW(g)	14.61±1.53^a^	15.90±0.89^b^	14.29±1.78	16.06±1.54^d^	18.67±1.31	17.15±1.54^e^	18.36±0.97	17.69±1.11^g^
Heart(mg)	109.21±24.35 ^a^	97.33±19.74^b^	111.52±24.65	106.92±36.50	132.83±33.56	112.6±16.84^e^	135.01±18.69	127.17±14.48
Lung(mg)	121.32±11.99 ^a^	123.81±15.44	124.57±10.49	126.82±13.78	139.62±11.34	134.81±17.39	138.28±10.13	131.11±14.51
Liver(mg)	1150.38±129.98 ^a^	1210.51±103.04^b^	1129.99±118.09	1272.92±149.75^d^	1085.1±118.12	1005.64±156.89^e^	1003.7±178.36^f^	1122.77±99.12
Spleen(mg)	194.89±23.45 ^a^	223.16±12.50^b^	212.73±23.48^c^	229.81±24.75^d^	126.26±32.79	115.42±38.67^e^	133.64±15.3	126.37±19.85
Kidney(mg)	290.8±32.22 ^a^	286.72±27.68	322.95±25.46^c^	299.84±37.48	374.87±34.59	368.07±17.44	368.21±24.64	353.79±43.91^g^
Heart/LBW(mg/g)	7.45±0.43	6.11±0.47	7.73±0.40	6.86±0.64	7.12±0.57	6.57±0.38	7.34±0.43	7.17±0.36

LBW: Lean body weight; Each point represents the mean ±SD.

^a^, Cancer group compared with normal control group;

^b^,DOX-treated tumor-bearing group compared with control cancer group;

^c^,DZR-treated tumor-bearing group compared with control cancer group;

^d^, DOX+DZR treated tumor-bearing group compared with control cancer group;

^e^, DOX-treated control group compared with normal control group;

^f^,DZR-treated control group compared with control group;

^g^, DOX+DZR-treated control group compared with normal control group

### DZR prevented DOX-induced myocardial injury: histopathology and biomarkers

**Fig 2:**
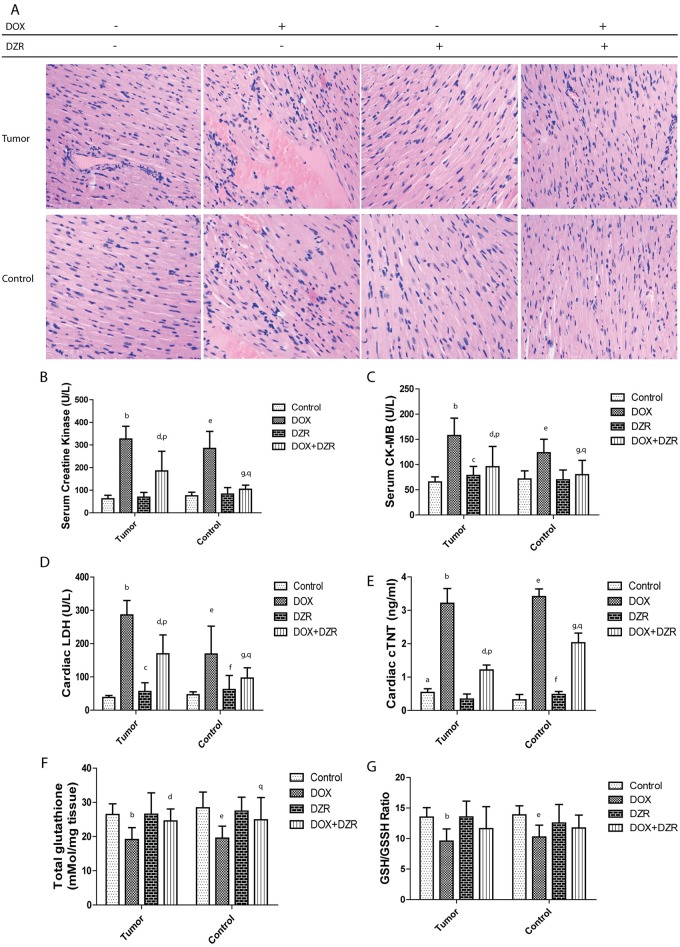
DZR protected against DOX-induced myocardial damage. Histopathology of HE staining of left ventricular walls indicated myocardial damage and cardioprotective effects of DZR(**A**). Magnification200x. DZR abrogated DOX-induced increase serum creatine kinase (**B**),CK-MB(**C**), cardiac LDH(D), cTNT(E), as well as decrease cardiac total glutathione (F) and GSH/GSSH ratio (G).

DOX-induced cardiac histopathological features included chronic inflammatory cell infiltration, interstitial edema, myocardial fibrosis, myocytolysis and myocardial necrosis in experimental animal models. In the present study, myocardial histopathology of HE staining from normal and cancer control mice showed normal architecture[24, 25]. However, DOX treatment resulted in chronic focal inflammatory pathology in the myocardium (Fig 2A). The inflammatory infiltrate reflected the replacement of degenerated myocardial muscle fibers with lymphocytes, and plasma cells. DZR-treated mice showed no histological changes in the heart tissues in the normal or cancer control group. The DOX+DZR treatment cancer group showed inflammatory infiltration. Further, the DOX+DZR control group showed normal heart tissues morphologically, without any inflammation or tissue injury. To evaluate the cardiac injury, we also determined serum-related markers (Fig 2B–2G). Results showed significantly increased serum creatine kinase and CK-MB in the DOX-treated group compared with the control. However, there was litter influence of DZR on these myocardial injury markers between control and the corresponding DZR-treated mice. Though the differences in serum CK-MB were significant, the increased concentration levels suggested no damage. Further, the DOX+DZR groups of mice showed significantly reduced serum and cardiac levels of these markers when compared with the DOX-treated group. LDH and cTNT were increased in the DOX-treated groups and alleviated in the DOX+DZR treated groups. There was no significant difference of GSSH, while total glutathione and ratio of GSH/GSSH was significant decreased after DOX treatment.

### Distinct metabolic profiling

**Fig 3:**
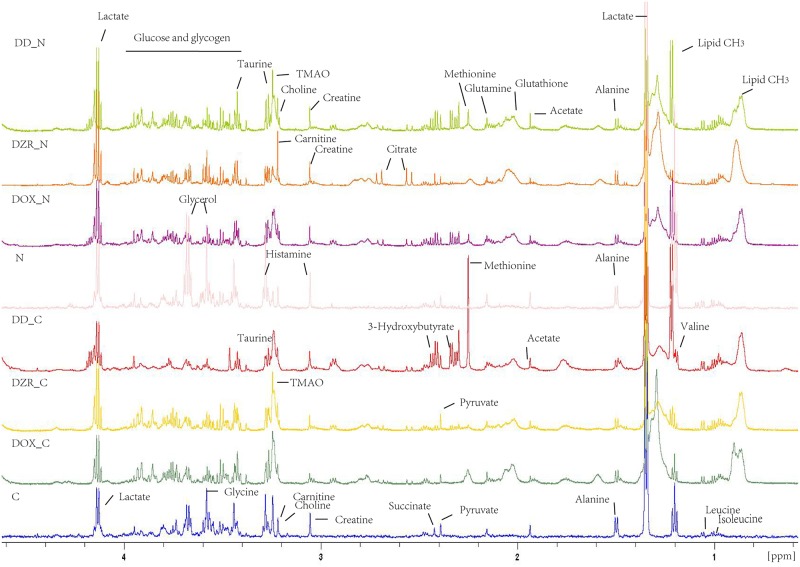
The typical 1H-NMR spectra of the serum from the different treatment models. The metabolites are assigned and marked. The overlapping peaks were identified by adding a reference substance to the sample.

**Fig 4:**
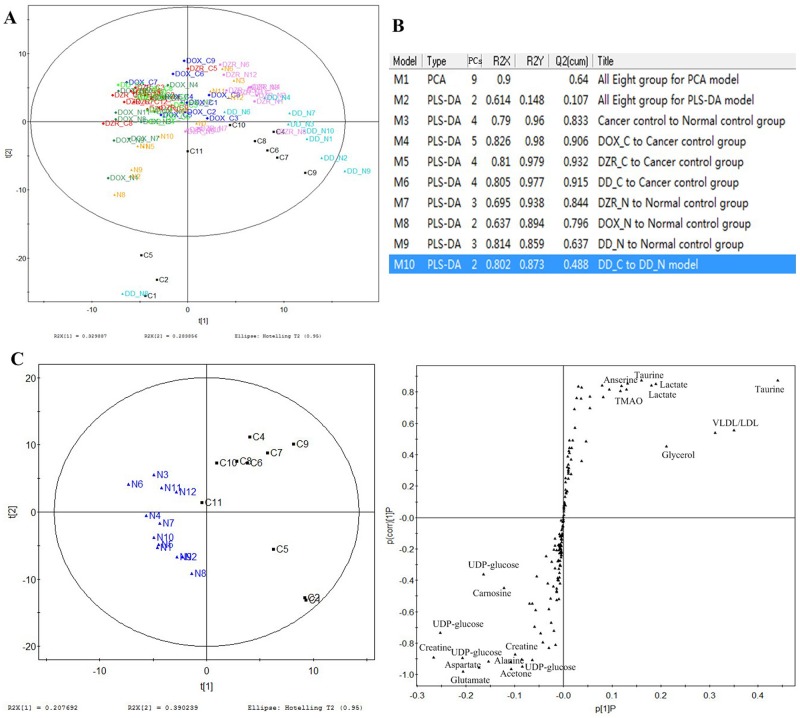
Overall profiling of the eight groups and abnormal metabolism in cancer. Score plot of the eight groups (A) shows treatment differences. The summary of pairwise metabolomics analysis of the model represents the cumulative R2X, R2Y and Q2 levels (**B**). The score plot and S-plot of the pairwise analysis of cancer and control groups reveal altered metabolites including creatine, UDP-glucose, VLDL/LDL, glycerol, TMAO, taurine, carnosine, lactate, acetone, glutamate, and aspartate (**C**).

The typical ^1^H NMR spectra of the serum demonstrated a characteristic metabolic profile along with metabolite assignments (Fig 3). The spectra contained very high-intensity signals from VLDL/LDL, alanine, lactate, acetate, pyruvate, creatine, and glucose(S1 Table). To illustrate the differences in metabolic profiles, the NMR spectra were further segmented and subjected to PCA. The score plot revealed distinct separation of tumor-bearing models from the control mice (Fig 4A). Further, the DOX-treated groups were clustered together, and the DZR-treated groups clustered in a different region. PLS-DA models were designed to identify the metabolites underlying the differences between the groups. Two PCs discriminated the overall metabolic profiling of the eight groups. Although the score plots confirmed the unique metabolic profiling in separate models, the low cumulative R^2^Y (0.148) and Q^2^ (0.107) challenged further analysis. Pairwise comparison was then used to recognize the metabolic profiling and the distinct metabolites. Pairwise comparison models from M3 to M9 explained more than 80% of the variation in Y, with excellent predictive ability (Q^2^(cum) was more than 63.7%) (Fig 4B).

### Cancer triggered glucose and lipid metabolic disorders

Pairwise comparison of cancer control and normal control groups reflected the distinct influence of cancer on mouse metabolism. The score plot of PC1 versus PC2 from the PLS-DA model in the pairwise comparison analysis of cancer and normal control groups showed an obvious distribution, indicating the characteristic metabolic profiling of cancer (Fig 4C). PC1 was the primary principal component in this separation, and S-plot from PC1 showed different metabolite signals. Based on the chemical shift in the spectrogram, along with the coupling constant and spitting pattern, the signals were identified and metabolites were marked. The metabolites with VIP>1 from PC1 represented distinct metabolite changes. Cancer triggered 17 changed metabolites, including decreasing creatine, UDP-glucose, VLDL/LDL, glycerol, anserine, 2-phosphoglycerate, 3-methylhistine, TMAO and taurine, as well as increasing carnosine, lactate, acetoacetate, glucose, glycylproline, acetone, glutamate, and aspartate. These metabolites were mainly derived from glucose and lipid, indicating altered glycolysis and lipid metabolism in cancer.

### DOX induced metabolic changes via enhanced oxidation

**Fig 5:**
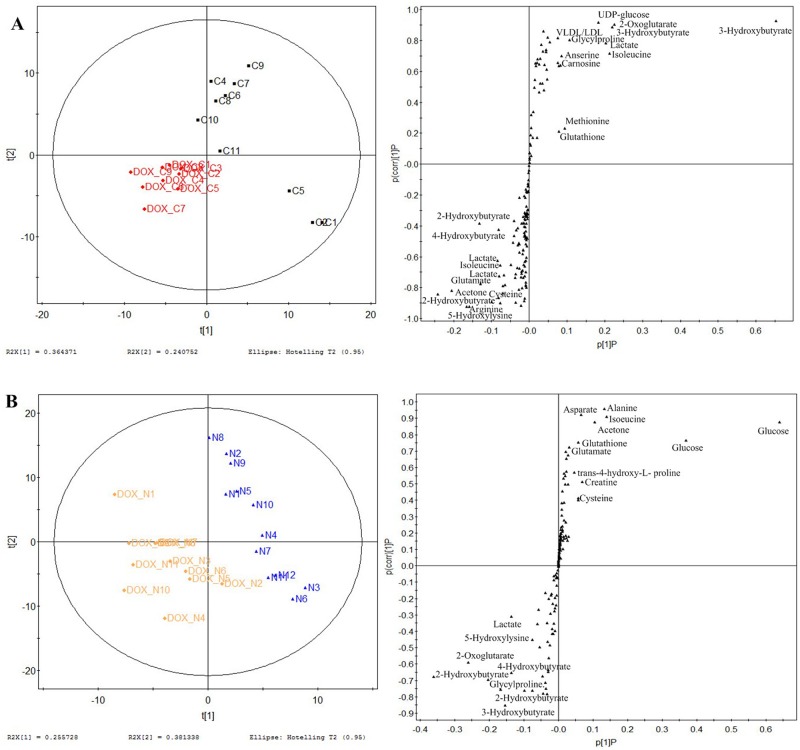
Two-paired PLS-DA score plot and S-plot revealed DOX-induced metabolic perturbations. The comparative analysis of DOX_C and cancer control groups reveal altered metabolite levels following DOX treatment of tumor-bearing mice (**A**), while the comparative analysis of DOX_N and normal control groups reveal distinct metabolic effects of DOX in normal animals (**B**).

Two pairwise comparisons were used to analyze the distinct metabolic effect of DOX. First, DOX-treated tumor-bearing mice were compared with control mice to show the effect of DOX on cancer. The score plot of PLS-DA model revealed the distinct serum metabolic profile of the two groups (Fig 5A). PC1 was also the primary principal component in this separation. The S-plot from PC1 of the model illustrated the altered metabolite levels following DOX treatment for cancer: increase in 2-oxoglutarate, VLDL/LDL,3-hydroxybutyrate, 5-hydroxylysine, 4-hydroxybutyrate, 2-hydroxybutyrate, creatine and arginine, as well as decrease in acetone, glutamate, lactate, cysteine, isoleucine, asparate, UDP-glucose, glycylproline, methionine, and carnosine levels. To eliminate the influence of cancer and focus on the unique effect of DOX, we compared DOX-treated control mice with normal controls. The score plot of the PLS-DA model revealed that PC1 discriminated between the two groups (Fig 5B). The S-plot revealed increased 4-hydroxybutyrate, trans-4-hydroxy-L- proline, 5-hydroxylysine, alanine, 2-hydroxybutyrate, 2-oxoglutarate, 3-hydroxybutyrate, creatine, and decrease in glucose, glutamate, acetoacetate, acetone, TMAO, cysteine, asparate, isoleucine, and glycylproline levels. Alteration in the common metabolites indicated a unique effect of DOX on metabolic regulation. The metabolite profiles following DOX treatment include increase in 4-hydroxybutyrate, 5-hydroxylysine, 2-hydroxybutyrate, 2-oxoglutarate, 3-hydroxybutyrate, and decrease of glucose, glutamate, cysteine, acetone, methionine, asparate, isoleucine, and glycylproline, suggesting enhanced oxidation. The classification and their variation tendency were list in S1 Table.

### DZR altered the metabolic profile of control and cancer mice

**Fig 6:**
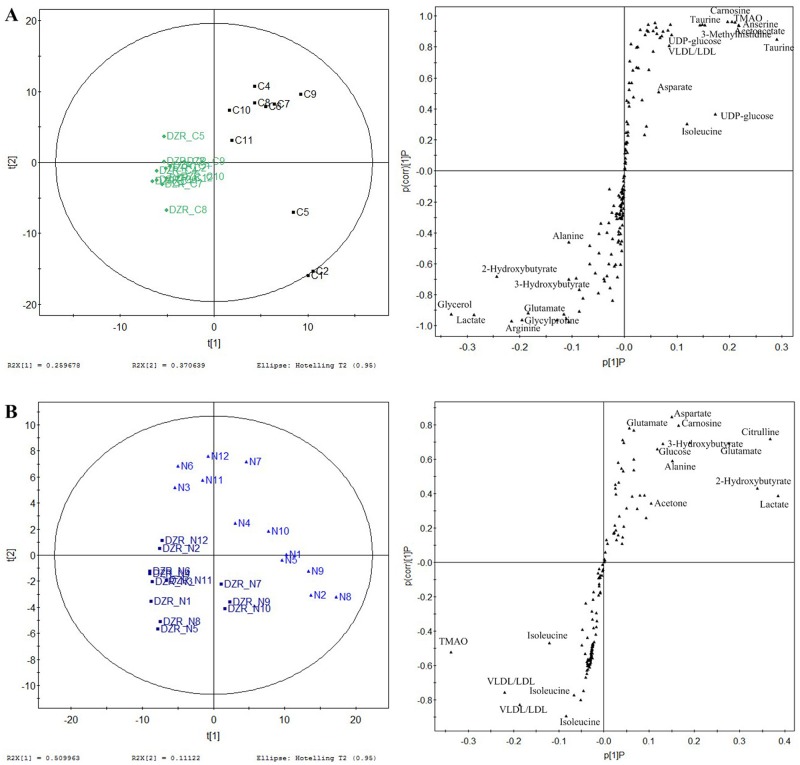
Two paired PLS-DA score and S-plot reveal altered metabolite levels following DZR treatment. The comparative analysis of DZR_C and cancer control groups revealed the altered metabolite levels resulting from DZR treatment of tumor-bearing mice (A), while the comparative analysis of DZR_N and normal control groups suggests distinct metabolic effect of DZR in normal animals.

We monitored the distinct metabolic response of DZR in cancer and normal mice based on two pairwise comparisons. First, we analyzed the different metabolic profiles of DZR-treated cancer mice with tumor-bearing mice. Score plots of PC1 versus PC2 from the PLS-DA model showed the differences while the S-Plot from PC1 showed different metabolic profiles (Fig 6A). DZR induced metabolite changes including substantial excretion of 3-hydroxybutyrate, glutamate, taurine, 3-methylhistine, lactate, anserine, glycerol, alanine, and arginine. Metabolites, such as UDP-glucose, TMAO, acetoacetate, isoleucine, asparate, and carnosine were decreased. Further, we characterized the distinct metabolic effect of DZR, by comparing the DZR-treated normal group with normal controls (Fig 6B)and found an increase in lactate, asparate, acetone, 3-hydroxybutyrate, glutamate, alanine, and decrease in glucose, VLDL/LDL, TMAO, citrulline, carnosine, and isoleucine. DZR treatment increased the levels of lactate, 3-hydroxybutyrate, glutamate, alanine, and decrease of glucose, TMAO and carnosine.

### DOX+DZR combination therapy resulted in distinct metabolic signatures

**Fig 7:**
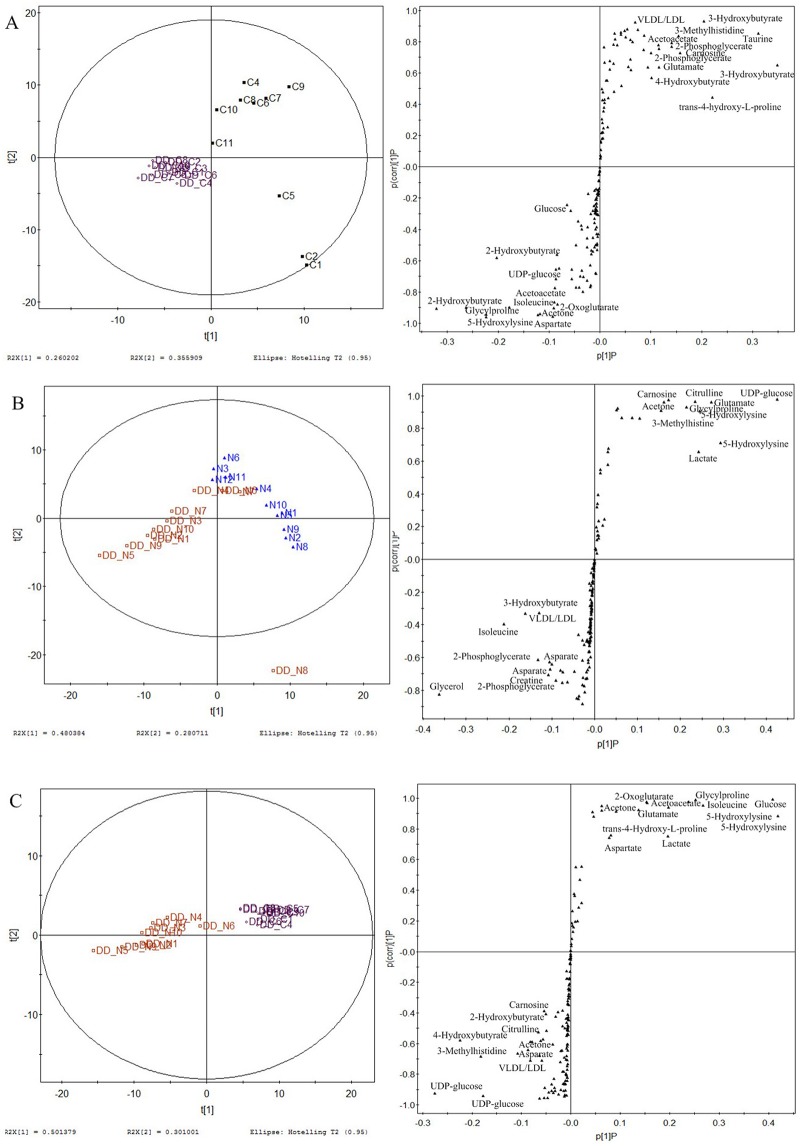
Three paired PLS-DA score plot and S-plot reveal altered metabolites following combined therapy with DOX and DZR. The comparative analysis of DD_C and DD_N groups indicates metabolic regulation by DOX + DZR (**A**), The comparative analysis of DD_C and cancer control groups indicates the metabolic regulation by DOX + DZR in cancerous mice (**B**), and the comparative analysis of DD_N and normal control groups reveals the unique metabolic effect of DOX and DZR on normal animals.

To illustrate the metabolic interaction of DOX and DZR, three pairwise comparisons were employed. First, we compared the DOX+DZR co-treatment cancer group with the control group (Fig 7A). We found increased levels of 5-hydroxylysine, 4-hydroxybutyrate, 2-oxoglutarate, VLDL/LDL, 3-hydroxybutyrate, 3-methylhistine, taurine, alanine, 2-hydroxybutyrate, anserine, 2-phosphoglycerate, UDP-glucose, trans-4-hydroxy-L-proline, and decrease of glucose, acetoacetate, glutamate, asparate, isoleucine, acetone, carnosine, and glycylproline. We compared DOX+DZR treated normal group with normal control group (Fig 7B), and found an increase in asparate, acetone, glutamate, glycylproline, isoleucine, lactate, acetoacetate, creatine, 3-hydroxybutyrate, and decrease in UDP-glucose, 5-hydroxylysine, 2-phosphoglycerate, glycerol, 3-methylhistine, VLDL/LDL, TMAO, citrulline, and carnosine. Finally, we compared DOX+DZR-treated cancer group with DOX+DZR-treated control group (Fig 7C). We found increased UDP-glucose, 4-hydroxybutyrate,5-hydroxylysine, carnosine, 2-oxoglutarate, trans-4-hydroxy-L-proline, citrulline, 2-hydroxybutyrate, VLDL/LDL, and alanine, as well as decreased levels of lactate, acetoacetate, glucose, 3-methylhistine, glutamate, isoleucine, glycylproline, asparate, andacetone. The metabolic changes induced by DOX+DZR treatment do not equal the sum of the altered metabolites following DOX and DZR treatment.

## Discussion

**Fig 8:**
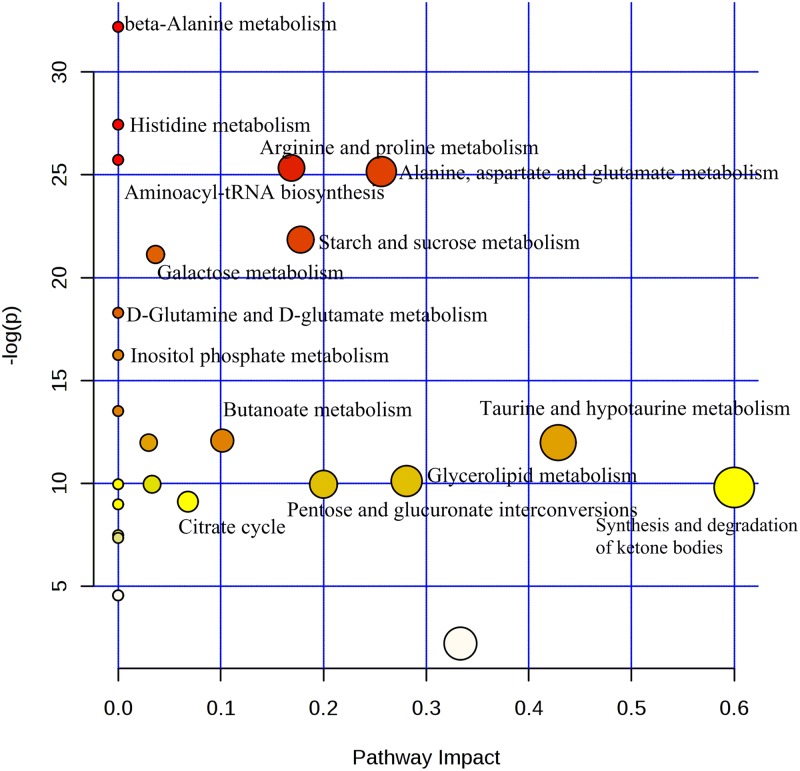
Cardioprotective effects of DZR on DOX-induced cardiotoxicity involving reprogrammed metabolic pathway.

The present study represents a metabolomics analysis of systemic variation following DOX and DZR treatment. The serum CK and CK-MB, as well as HE staining confirmed DOX-induced myocardial damage and DZR-related cardioprotective effects. The metabolic effects of DOX and DZR were distinguishable in serum ^1^H NMR based metabolomics profiling. We identified a panel of altered metabolites. Based on the changed metabolites, the potential metabolic pathways were established (Fig 8). In addition to synthesis and degradation of ketone bodies, alanine and histidine metabolism represented significant pathways(S2 Table).

The quinine functional groups of DOX were activated by various reductases to free semiquinone structures. The semiquinone was bound to iron and catalyzed the formation of toxic and reactive oxygen species, such as hydroxyl radical, superoxide anion and hydrogen peroxide [26, 27]. The DOX-induced oxidative stress led to mitochondrial dysfunction. Mitochondria dysfunction resulted in mitochondrial iron deficiency and increase in cellular iron levels. The increased iron in the cytoplasm generated reactive oxygen species, contributing to metabolite aberrations, which are the main factors contributing to myocardial toxicity of DOX [28]. DZR is a bisdioxopiperazine that easily enters cells, followed by hydrolysis into an ethylenediaminetetraacetic acid analog, and serving as a strong iron chelator [29]. It potentially displaces iron and prevents cardiotoxicity associated with DOX[12]. As expected, we found increased levels of hydroxyl radical metabolites, such as 4-hydroxybutyrate, 5-hydroxylysine, 2-hydroxybutyrate, and 3-hydroxybutyrate, in DOX-treated groups. DOX-induced cardiotoxicity was mainly attributed to increased myocardial oxidative stress. The acidic character of 3-hydroxybutyrate decreased the oxidative damage [30]. Further, 3-hydroxybutyrate activated pentose phosphate pathway and increased NADPH synthesis, which is a key cofactor in the activity of various anti-oxidative enzymes [31]. These results indicated that 3-hydroxybutyrate protected against DOX-induced oxidative stress. Interestingly, in the present study, 3-hydroxybutyrate was also elevated in the DZR-treated groups. Treatment with DOX combined with DZR upregulated cellular 3-hydroxybutyrate levels. In mammals, the rate-limiting enzyme 3-hydroxybutyrate dehydrogenase catalyzes the biogenesis of small iron-binding molecules that facilitate iron uptake[30]. Inhibition of 3-hydroxybutyrate dehydrogenase results in abnormal accumulation of intracellular iron, mitochondrial iron deficiency and increased oxidative stress.

Excessive oxidant levels are normally scavenged by glutathione and its intermediate metabolites [32, 33]. In the present study, glutathione was decreased in the DOX treatment group. Glutathione depletion greatly exacerbated DOX-induced cardiotoxicity [34], and increased glutathione was insufficient to protect against DOX-induced myocardial damage[32]. In our study, there was no significant difference of GSSH, while total glutathione was decreased in the DOX- treated group when compared with control group. The ratio of GSH/GSSH was significant decreased after DOX treatment, and DZR increased the ratio of GSH/GSSH from9.57 to 11.62. Moreover, DZR treatment increased serum glutamate levels, resulting in cardioprotective effects. Additionally, the dramatically decreased serum cysteine and its precursor methionine suggested the increased role of antioxidants against elevated oxidative stress following DOX treatment. Higher serum concentrations of cysteine were significantly associated with a lower risk of DOX-related cardiotoxicity [35]. The persistently decreased cysteine level in the DOX-treated groups suggests a progressive oxidative stress indicating DOX-induced cardiotoxicity. DZR had no role in the metabolism of cysteine and methionine.

Arginine and citrulline were also decreased in the DOX-treated group. The two amino acids involved in the urea cycle exert multiple cardioprotective effects. They were particularly important during periods of acute myocardial injury, as these states tend to be characterized as increasing degrading enzyme arginase, resulting in a transient arginine deficiency [36, 37]. Further, arginine represents a by-product of nitric oxide formation. A previous study showed that nitric oxide levels were decreased in DOX-induced congestive heart failure suggesting the possible role of plasma nitric oxide levels in DOX-induced cardiotoxicity[38, 39]. In the present metabolomics study, we found serum citrulline level was decreased in DZR treatment group and increased in the DOX+ DZR treatment group. Arginine was increased in the DOX treatment group and combined treatment group. These was consistent with previous study that the ratio of citrulline to arginine was increased in DOX mono-treatment group and decreased in the combined treatment of DZR and DOX.

Previous transcriptomics study showed that energy metabolism and apoptosis was significantly altered before and after occurrence of myocardial injury after DOX treatment[40]. Transcriptional changes were the upstream regulatory and metabolites could give an instantaneous snapshot of the physiology and pathological state. Our metabolomics study validated the altered energy metabolic profile. Moreover, it was interesting that much of these DOX-induced transcriptional changes were attenuated by pretreatment of mice with DZR. These was agree with previous study that DZR prevented DOX induced cardiomyopathy and protected the cardiac mitochondria from acquired functional damage[9].

In conclusion, the present study represents a metabolomics approach to elucidate DOX-induced cardiotoxicity and the reprogramming effects of DZR. The results suggest DOX-induced cardiotoxicity and DZR-related cardioprotection. In addition, the metabolites represent potential biomarkers for prognostic evaluation of cardiotoxicity induced by DOX and cardioprotection by DZR. Additional studies are needed to corroborate the findings of the current *in vivo* murine model.

## Supporting Information

S1 Fig Change of tumor volume (cm3) over the course of your experiment. (DOCX)

S1 Table The identification, assignment, origin of metabolites and their variation. (DOCX)

S2 Table Analysis of the altered metabolic pathways resulted from DOX+DZR treatment. The table below shows the detailed results from the pathway analysis. Since we are testing many pathways at the same time, the statistical p values from enrichment analysis are further adjusted for multiple testing. In particular, the Total is the total number of compounds in the pathway; the Hits is the actually matched number from the user uploaded data; the Raw p is the original p value calculated from the enrichment analysis; the Holm p is the p value adjusted by Holm-Bonferroni method; the FDR p is the p value adjusted using False Discovery Rate; the Impact is the pathway impact value calculated from pathway topology analysis.
(DOCX)
