# Supplementary figures and images for: Protective Effects of Dexrazoxane against Doxorubicin-Induced Cardiotoxicity: A Metabolomic Study
Source: PLoS One. 2017 Jan 10;12(1):e0169567. doi: 10.1371/journal.pone.0169567 (PMC5224977; doi:10.1371/journal.pone.0169567)

S1 Fig. Change of tumor volume (cm3) over the course of your experiment


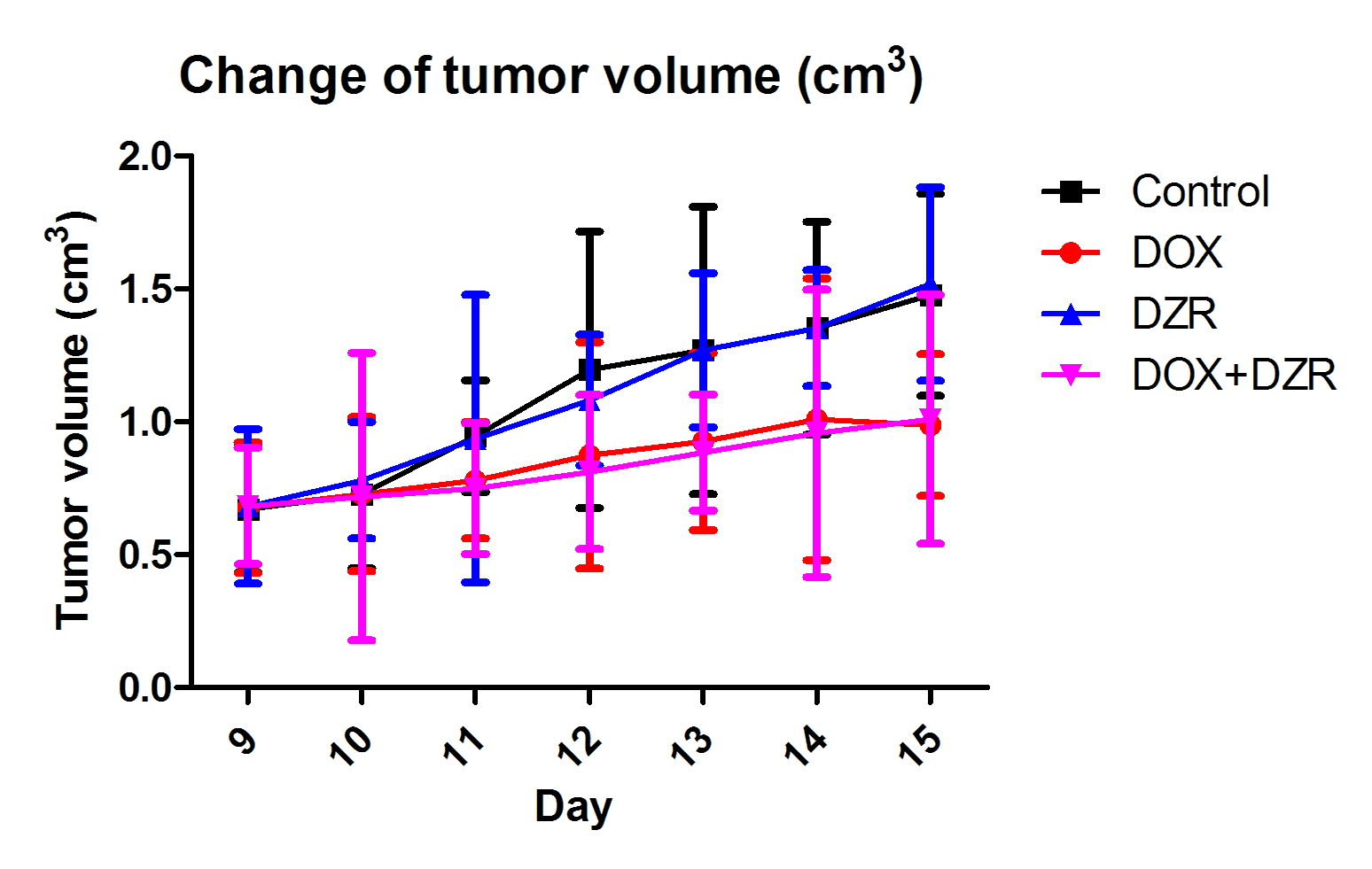

Supplement: S1 Fig — (DOCX) [file pone.0169567.s001.docx]
