# Supplementary material for: Protective Effects of Dexrazoxane against Doxorubicin-Induced Cardiotoxicity: A Metabolomic Study
Source: PLoS One. 2017 Jan 10;12(1):e0169567. doi: 10.1371/journal.pone.0169567 (PMC5224977; doi:10.1371/journal.pone.0169567)
Supplement: S1 Table — (DOCX) [file pone.0169567.s002.docx]

S1 Table. The identification, assignment, origin of metabolites and their variation

| Metabolites | δ1H (multiplicityb) | Category | N | C | DOX_N | DOX_C | DZR_N | DZR_C | DD_N | DD_C |
| --- | --- | --- | --- | --- | --- | --- | --- | --- | --- | --- |
| 2-Hydroxybutyrate | 4.00(m), 0.88(t) | Fatty acids and conjugates | 1.00±0.11 | 0.89±0.32 | 1.66±0.23 | 1.59±0.30 | 1.00±0.34 | 1.80±0.16 | 0.69±0.28 | 1.72±0.16 |
| 2-oxoglutarate | 3.03(t) | Gamma-keto acids and derivatives | 1.00±0.19 | 1.02±0.46 | 1.65±1.29 | 5.06±3.14 | 1.37±0.37 | 1.84±1.90 | 0.94±0.56 | 3.05±2.22 |
| 2-Phosphoglycerate | 3.81(s), 3.90(s), 4.47(m) | Sugar acids and derivatives | 1.00±0.20 | 0.56±0.13 | 1.07±0.25 | 0.88±0.23 | 1.06±0.16 | 1.28±0.17 | 0.62±0.30 | 1.03±0.28 |
| 3-Hydroxybutyrate | 1.24 (t), 3.69 (q) | Fatty acids and conjugates | 1.00±0.18 | 1.29±0.56 | 1.56±1.18 | 4.62±2.74 | 1.54±0.28 | 1.92±1.86 | 1.61±0.49 | 2.75±1.99 |
| 3-Methylhistine | 7.09 (s), 7.78 (s) | Aralkylamines | 1.00±0.20 | 0.46±0.17 | 0.99±0.29 | 0.69±0.24 | 1.04±0.24 | 1.11±0.19 | 0.59±0.28 | 0.96±0.32 |
| 4-Hydroxybutyrate | 3.59(t), 2.22(t),1.79(m) | Short-chain hydroxy acids derivatives | 1.00±0.24 | 1.02±0.64 | 3.47±1.04 | 3.04±0.37 | 0.97±0.54 | 3.09±0.34 | 0.68±0.45 | 3.18±0.42 |
| 5-hydroxylysine | 3.77(m), 3.88(m), 2.93(m), 3.16(Br) | Fatty acids and conjugates | 1.00±0.32 | 0.76±0.31 | 2.83±0.82 | 2.55±0.39 | 0.59±0.30 | 2.35±0.25 | 0.63±0.54 | 2.72±0.34 |
| Acetoacetate | 3.27(s),3.44(s) | Short-chain keto acids derivatives | 1.00±0.15 | 1.59±0.93 | 0.83±0.46 | 1.72±0.67 | 1.05±0.28 | 0.92±0.46 | 1.84±0.89 | 1.02±0.38 |
| Acetone | 2.23(s) | Ketones | 1.00±0.19 | 1.90±0.80 | 0.78±0.38 | 1.17±0.39 | 1.56±0.45 | 0.70±0.19 | 2.84±1.08 | 0.77±0.19 |
| Alanine | 1.50 (d), 3.80 (q) | alpha-amino acids | 1.00±0.20 | 1.09±0.37 | 2.32±0.31 | 1.89±0.47 | 1.21±0.71 | 2.19±0.23 | 1.03±0.38 | 2.16±0.27 |
| Anserine | 3.76(s),7.10(S),8.25(s) | Amino acids | 1.00±0.18 | 0.51±0.19 | 1.01±0.15 | 0.90±0.17 | 0.99±0.14 | 1.10±0.14 | 0.69±0.30 | 0.97±0.21 |
| Arginine | 1.89(m), 3.24(t), 3.77(t) | alpha-amino acids | 1.00±0.09 | 0.77±0.38 | 1.23±0.16 | 1.38±0.23 | 1.04±0.21 | 1.43±0.13 | 0.68±0.27 | 1.31±0.12 |
| Asparate | 2.85(dd), 2.94(dd), 4.00(q) | Carboxylic acids and derivatives | 1.00±0.19 | 1.85±0.75 | 0.75±0.13 | 0.80±0.26 | 1.77±0.36 | 0.65±0.11 | 2.96±1.12 | 0.80±0.12 |
| Carnosine | 3.21(m),3.66(m),4.46(m),7.08(s),8.11(s) | Carboxylic acids and derivatives | 1.00±0.80 | 2.71±3.39 | 1.07±0.30 | 0.57±0.22 | 0.39±0.38 | 0.83±0.26 | 0.22±0.14 | 0.83±0.10 |
| Citrulline | 3.03 (s), 3.91 (s) | alpha-amino acids | 1.00±0.36 | 0.70±0.37 | 0.96±0.26 | 0.87±0.18 | 0.60±0.23 | 0.86±0.21 | 0.38±0.18 | 0.97±0.21 |
| Creatine | 3.04 (s), 4.10 (s) | Carboxylic acids and derivatives | 1.00±0.11 | 0.75±0.29 | 1.20±0.15 | 1.18±0.26 | 1.00±0.13 | 1.28±0.15 | 0.66±0.23 | 1.25±0.16 |
| Glucose | 4.69 (d), 3.74 (dd), 3.91 (dd), 5.27 (d) | Carbohydrates and conjugates | 1.00±0.26 | 1.57±0.72 | 0.87±0.26 | 0.63±0.16 | 0.95±0.19 | 0.78±0.15 | 1.35±0.35 | 0.68±0.11 |
| Glucose-6-phosphate | 5.22(d), 4.64(d) | Carbohydrates and conjugates | 1.00±0.50 | 5.03±7.66 | 4.71±2.41 | 4.17±0.54 | 1.00±0.94 | 3.92±0.86 | 0.79±0.85 | 4.64±0.94 |
| Glutamate | 2.06(m), 2.34(m) | Carboxylic acids and derivatives | 1.00±0.16 | 1.86±0.91 | 0.84±0.15 | 1.03±0.26 | 1.41±0.29 | 0.98±0.14 | 2.12±0.95 | 0.83±0.12 |
| Glycerol | 3.81 (m), 3.71(m), 3.69 (m) | Carbohydrates and conjugates | 1.00±0.18 | 0.49±0.12 | 0.94±0.31 | 0.58±0.27 | 1.05±0.23 | 1.02±0.23 | 0.59±0.29 | 0.85±0.36 |
| Glycylproline | 3.95(s) | Carboxylic acids and derivatives | 1.00±0.17 | 1.95±0.93 | 0.54±0.25 | 0.75±0.17 | 1.18±0.31 | 0.57±0.16 | 1.97±0.71 | 0.56±0.17 |
| Homocitrulline | 3.73(t), 1.86(m) | Carboxylic acids and derivatives | 1.00±0.12 | 0.88±0.34 | 1.30±0.24 | 1.41±0.3 | 1.06±0.23 | 1.48±0.16 | 0.73±0.27 | 1.52±0.19 |
| isoleucine | 0.98 (t), 1.01 (d), 1.28 (m), 1.49 (m), 1.98 (m), | Branch chain amino acids | 1.00±0.18 | 1.60±0.99 | 0.66±0.14 | 0.81±0.24 | 1.14±0.27 | 0.66±0.10 | 1.86±0.92 | 0.69±0.10 |
| Lactate | 1.33 (d), 4.14 (q) | Alpha hydroxy acids and derivatives | 1.00±0.46 | 2.14±0.81 | 1.24±0.28 | 1.18±0.53 | 1.91±2.00 | 1.48±0.37 | 1.86±0.92 | 1.23±0.21 |
| leucine | 0.94 (d), 1.00 (d), 1.65 (m), 1.70 (m), 3.73 (m) | Branch chain amino acids | 1.00±0.13 | 0.90±0.35 | 0.96±0.12 | 1.05±0.10 | 0.98±0.14 | 1.11±0.09 | 1.06±0.22 | 1.11±0.20 |
| VLDL/LDL | 1.31 (m), 1.61 (m), 2.06 (m), 2.26 (m), 2.77 (m) | Lipid | 1.00±1.02 | 0.56±0.29 | 0.86±0.42 | 2.21±1.09 | 0.83±0.37 | 0.85±0.68 | 0.56±0.23 | 1.38±0.88 |
| myo-Inositol | 3.53(dd), 3.62 (t) | Cyclic alcohols and derivatives | 1.00±0.22 | 1.21±0.40 | 1.35±0.18 | 1.32±0.30 | 1.12±0.63 | 1.51±0.13 | 1.11±0.21 | 1.41±0.12 |
| Pyruvate | 2.31 (s) | Alpha-keto acids and derivatives | 1.00±0.40 | 1.58±0.42 | 0.89±0.21 | 0.80±0.20 | 0.86±0.42 | 0.77±0.18 | 1.44±0.59 | 0.94±0.22 |
| Serine | 3.99 (m), 4.01 (m), 3.85 (m) | Carboxylic acids and derivatives | 1.00±0.19 | 0.56±0.19 | 1.05±0.27 | 0.83±0.24 | 1.13±0.19 | 1.23±0.18 | 0.64±0.30 | 1.01±0.31 |
| Taurine | 3.26 (t), 3.43(t) | Sulfonic acids | 1.00±0.23 | 0.44±0.17 | 0.98±0.25 | 0.75±0.23 | 1.05±0.21 | 1.09±0.20 | 0.68±0.31 | 0.90±0.34 |
| trans-4-Hydroxy-L-proline | 4.67(m),4.34(m),3.36(m),3.47(m) | Carboxylic acids and derivatives | 1.00±0.24 | 1.64±2.86 | 3.14±0.96 | 2.51±0.44 | 1.18±0.59 | 2.76±0.29 | 0.98±0.99 | 3.00±0.48 |
| Trimethylamine N-oxide | 3.26 (t) | Amine oxides and derivatives | 1.00±0.20 | 0.45±0.21 | 0.77±0.11 | 0.76±0.18 | 0.79±0.15 | 0.79±0.14 | 0.51±0.24 | 0.83±0.14 |
| UDP-glucose | 4.37(m), 5.60(m) | Carbohydrates and conjugates | 1.00±0.20 | 0.56±0.22 | 1.07±0.23 | 0.90±0.24 | 1.14±0.18 | 1.25±0.17 | 0.63±0.33 | 1.03±0.29 |
