# Supplementary material for: Protective Effects of Dexrazoxane against Doxorubicin-Induced Cardiotoxicity: A Metabolomic Study
Source: PLoS One. 2017 Jan 10;12(1):e0169567. doi: 10.1371/journal.pone.0169567 (PMC5224977; doi:10.1371/journal.pone.0169567)
Supplement: S2 Table — The table below shows the detailed results from the pathway analysis. Since we are testing many pathways at the same time, the statistical p values from enrichment analysis are further adjusted for multiple testing. In particular, the Total is the total number of compounds in the pathway; the Hits is the actually matched number from the user uploaded data; the Raw p is the original p value calculated from the enrichment analysis; the Holm p is the p value adjusted by Holm-Bonferroni method; the FDR p is the p value adjusted using False Discovery Rate; the Impact is the pathway impact value calculated from pathway topology analysis. (DOCX) [file pone.0169567.s003.docx]

S2 Table. Analysis of the altered metabolic pathways resulted from DOX+DZR treatment

| Pathway Name | Total | Hits | p | -log(p) | Holm p | FDR | Impact | Details |
| --- | --- | --- | --- | --- | --- | --- | --- | --- |
| beta-Alanine metabolism | 17 | 2 | 1.05E-14 | 32.191 | 2.62E-13 | 2.62E-13 | 0 | http://www.genome.jp/kegg-bin/show_pathway?mmu00410 |
| Histidine metabolism | 15 | 2 | 1.21E-12 | 27.439 | 2.91E-11 | 1.52E-11 | 0 | http://www.genome.jp/kegg-bin/show_pathway?mmu00340 |
| Aminoacyl-tRNA biosynthesis | 69 | 3 | 6.73E-12 | 25.725 | 1.55E-10 | 5.61E-11 | 0 | http://www.genome.jp/kegg-bin/show_pathway?mmu00970 |
| Arginine and proline metabolism | 44 | 5 | 9.95E-12 | 25.334 | 2.19E-10 | 5.93E-11 | 0.16882 | http://www.genome.jp/kegg-bin/show_pathway?mmu00330 |
| Alanine, aspartate and glutamate metabolism | 24 | 2 | 1.19E-11 | 25.158 | 2.49E-10 | 5.93E-11 | 0.25633 | http://www.genome.jp/kegg-bin/show_pathway?mmu00250 |
| Starch and sucrose metabolism | 19 | 2 | 3.24E-10 | 21.85 | 6.48E-09 | 1.35E-09 | 0.17773 | http://www.genome.jp/kegg-bin/show_pathway?mmu00500 |
| Galactose metabolism | 26 | 3 | 6.68E-10 | 21.127 | 1.27E-08 | 2.39E-09 | 0.03644 | http://www.genome.jp/kegg-bin/show_pathway?mmu00052 |
| D-Glutamine and D-glutamate metabolism | 5 | 2 | 1.14E-08 | 18.292 | 2.05E-07 | 3.56E-08 | 0 | http://www.genome.jp/kegg-bin/show_pathway?mmu00471 |
| Propanoate metabolism | 20 | 1 | 8.85E-08 | 16.24 | 1.5E-06 | 2.46E-07 | 0 | http://www.genome.jp/kegg-bin/show_pathway?mmu00640 |
| Inositol phosphate metabolism | 28 | 1 | 1.35E-06 | 13.515 | 2.16E-05 | 3.38E-06 | 0 | http://www.genome.jp/kegg-bin/show_pathway?mmu00562 |
| Butanoate metabolism | 22 | 3 | 5.68E-06 | 12.079 | 8.52E-05 | 1.2E-05 | 0.10145 | http://www.genome.jp/kegg-bin/show_pathway?mmu00650 |
| Taurine and hypotaurine metabolism | 8 | 1 | 6.26E-06 | 11.981 | 8.77E-05 | 1.2E-05 | 0.42857 | http://www.genome.jp/kegg-bin/show_pathway?mmu00430 |
| Primary bile acid biosynthesis | 46 | 1 | 6.26E-06 | 11.981 | 8.77E-05 | 1.2E-05 | 0.02976 | http://www.genome.jp/kegg-bin/show_pathway?mmu00120 |
| Glycerolipid metabolism | 18 | 1 | 4.05E-05 | 10.113 | 0.000487 | 6.97E-05 | 0.28098 | http://www.genome.jp/kegg-bin/show_pathway?mmu00561 |
| Pentose and glucuronate interconversions | 16 | 1 | 4.74E-05 | 9.9575 | 0.000521 | 6.97E-05 | 0.2 | http://www.genome.jp/kegg-bin/show_pathway?mmu00040 |
| Amino sugar and nucleotide sugar metabolism | 37 | 1 | 4.74E-05 | 9.9575 | 0.000521 | 6.97E-05 | 0.03306 | http://www.genome.jp/kegg-bin/show_pathway?mmu00520 |
| Ascorbate and aldarate metabolism | 9 | 1 | 4.74E-05 | 9.9575 | 0.000521 | 6.97E-05 | 0 | http://www.genome.jp/kegg-bin/show_pathway?mmu00053 |
| Synthesis and degradation of ketone bodies | 5 | 2 | 5.53E-05 | 9.8024 | 0.000521 | 7.68E-05 | 0.6 | http://www.genome.jp/kegg-bin/show_pathway?mmu00072 |
| Citrate cycle (TCA cycle) | 20 | 1 | 0.00011 | 9.1116 | 0.000773 | 0.000145 | 0.06799 | http://www.genome.jp/kegg-bin/show_pathway?mmu00020 |
| Tyrosine metabolism | 44 | 1 | 0.000126 | 8.9814 | 0.000773 | 0.000157 | 0 | http://www.genome.jp/kegg-bin/show_pathway?mmu00350 |
| Valine, leucine and isoleucine degradation | 38 | 2 | 0.000563 | 7.4829 | 0.002813 | 0.00067 | 0 | http://www.genome.jp/kegg-bin/show_pathway?mmu00280 |
| Glycine, serine and threonine metabolism | 31 | 1 | 0.000644 | 7.3476 | 0.002813 | 0.000732 | 0 | http://www.genome.jp/kegg-bin/show_pathway?mmu00260 |
| Glycolysis or Gluconeogenesis | 26 | 1 | 0.010507 | 4.5557 | 0.031522 | 0.010945 | 0 | http://www.genome.jp/kegg-bin/show_pathway?mmu00010 |
| Pyruvate metabolism | 23 | 1 | 0.010507 | 4.5557 | 0.031522 | 0.010945 | 0 | http://www.genome.jp/kegg-bin/show_pathway?mmu00620 |
| Valine, leucine and isoleucine biosynthesis | 11 | 1 | 0.10912 | 2.2153 | 0.10912 | 0.10912 | 0.33333 | http://www.genome.jp/kegg-bin/show_pathway?mmu00290 |

The table below shows the detailed results from the pathway analysis. Since we are testing manypathways at the same time, the statistical p values from enrichment analysis are further adjusted formultiple testings. In particular, the Total is the total number of compounds in the pathway; the Hits isthe actually matched number from the user uploaded data; the Raw p is the original p value calculatedfrom the enrichment analysis; the Holm p is the p value adjusted by Holm-Bonferroni method; theFDR p is the p value adjusted using False Discovery Rate; the Impact is the pathway impact valuecalculated from pathway topology analysis.
